# Editorial: Phytochemicals: molecular mechanisms for anti-aging and aging-related diseases

**DOI:** 10.3389/fnut.2026.2011625

**Published:** 2026-10-30

**Authors:** Chenghao Lv, Nattaya Konsue, Si Qin

**Affiliations:** 1 Hunan Provincial Key Laboratory of Liver Visceral Manifestation in Traditional Chinese Medicine, Institute of Integrative Medicine, Department of Integrated Traditional Chinese and Western Medicine, Xiangya Hospital, Central South University, Changsha, China; 2 School of Agro-Industry, Mae Fah Luang University, Muang Chiang Rai, Thailand; 3 Laboratory of Food Function and Nutrigenomics, College of Food Science and Technology, Hunan Agricultural University, Changsha, China

**Keywords:** aging, dietary intervention, evidence-based nutrition, oxidative stress, phytochemicals

Aging is a multisystem biological process driven by oxidative stress, mitochondrial dysfunction, impaired proteostasis, chronic inflammation, and metabolic dysregulation. With the global population aged 60 years and older projected to exceed 2 billion by 2050, identifying safe, effective, and translatable nutritional interventions has become a public-health priority. Food-derived bioactive compounds, including flavonoids, proanthocyanidins, iridoids, lignans, phytosterols, and triterpenoid saponins, offer considerable potential for extending healthspan and preventing or delaying age-related diseases. The present Research Topic brings together five published articles that span approaches from green extraction and phytochemical profiling to mechanism discovery, potential disease-specific applications, and population-based evidence.

The Research Topic spans complementary models and methodologies. Zhang J. et al. evaluated a phytochemical-rich *Eucommia ulmoides* leaf extract (EULE) in *Caenorhabditis elegans* to assess its effects on lifespan and healthspan and explore the underlying mechanisms. EULE was rich in bioactive compounds such as flavonoids, iridoids, and lignans. EULE supplementation extended lifespan and improved healthspan, including attenuation of age-related muscle deterioration, preservation of intestinal barrier integrity, and restoration of mitochondrial homeostasis. Transcriptomic analysis mitogen-activated protein kinase (MAPK) signaling, with validation pointing to the pmk-1/p38 MAPK pathway as a key mediator of anti-aging effects in this model.

Moving to cellular hepatocyte senescence, He et al. developed a green extraction platform for lotus peel proanthocyanidins using natural deep eutectic solvents (NADES). Under ultrasound-assisted conditions optimized by response surface methodology, the proanthocyanidin yield obtained with glycerol-betaine (1:1) was higher than that obtained with 70% ethanol. Standardized *in vitro* digestion revealed distinct release and stability profiles for procyanidins B2, B3, and B4. Procyanidin B4 retained 55.32% after 4 h of intestinal digestion and showed the strongest anti-senescence activity among the tested treatments in tert-butyl hydroperoxide (t-BHP)-treated human hepatoma-derived HepG2 cells. At 150 μg/mL, Procyanidin B4 reduced senescence-associated β-galactosidase (SA-β-gal)-positive cells and modulated the nuclear factor erythroid 2-related factor 2 (Nrf2)/Kelch-like ECH-associated protein 1 (Keap1)/antioxidant response element (ARE) pathway, increasing the activities of superoxide dismutase (SOD), catalase (CAT), and glutathione peroxidase (GSH-Px) while lowering reactive oxygen species (ROS) and malondialdehyde (MDA). This work links sustainable extraction, digestive stability, and redox-related anti-senescence effects.

For skin aging, Jia et al. integrated network pharmacology, a triple machine-learning strategy [support vector machine-recursive feature elimination (SVM-RFE), random forest, and least absolute shrinkage and selection operator (LASSO)], and experimental validation to investigate the anti-photoaging effects of dissect medicinal-edible black soybean. Among 63 overlapping targets, heat shock protein 90 alpha family class A member 1 (HSP90AA1) and B-cell lymphoma 2 (BCL2) emerged as core nodes. Molecular docking showed high-affinity binding of β-sitosterol to HSP90AA1 via isoleucine residue 214(ILE-214) in its client-binding region. In ultraviolet A (UVA)-irradiated human skin fibroblasts (HSFs), β-sitosterol up-regulated HSP90AA1, stabilized BCL2 and p53, reduced SA-β-gal positivity. The activities of antioxidant enzymes such as SOD, GSH-Px, and CAT increased, while the level of MDA decreased. Inhibition of HSP90AA1 abolished these benefits, supporting a role for the HSP90AA1-p53-BCL2 axis in this cellular photoaging model.

A disease-focused perspective is provided by Cen et al. on gypenosides from *Gynostemma pentaphyllum* for age-related macular degeneration (AMD). The authors synthesize preclinical evidence that gypenosides suppress ROS and MDA, enhance glutathione and Nrf2-mediated antioxidant defense, inhibit nuclear factor kappa B (NF-κB)-mediated inflammation, reduce caspase-dependent apoptosis in retinal pigment epithelial cells, and regulate cholesterol efflux and gut-microbiome–derived metabolites. They propose validation in high-fat/high-glucose and superoxide dismutase 1 (*Sod1*)-deficient AMD models, highlighting the convergence of oxidative, lipid-metabolic, and microbial mechanisms in retinal aging.

Finally, linking nutrition to geriatric outcomes, Zhang R. et al. studied the relationship between berry intake and frailty among 357 elderly inpatients in China and 5,553 adults over 65 years old in the United States, using National Health and Nutrition Examination Survey (NHANES) data from 2003 to 2018 for the US sample. Berry consumption was associated with lower odds of frailty after multivariable adjustment. In NHANES, significant associations were observed at 37–74 g/day and 74–158 g/day intakes; these findings do not indicate an association at all higher intakes. The mediation analysis of six blood markers revealed that glycated hemoglobin (HbA1c) was associated with berries and frailty. This supports the idea that the combined effect of blood sugar control, along with lipids, albumin, creatinine, uric acid, and triglycerides, plays a crucial role in connecting fruits rich in bioactive substances with the prevention of frailty.

Collectively, these articles reveal several cross-cutting themes. Firstly, the diversity of plant compounds is important: flavonoids, proanthocyanidins, isoflavones, lignans, β-sitosterol and gypenosides may act on different but overlapping anti-aging networks. Secondly, redox and stress response signaling pathways feature prominently, particularly Nrf2/Keap1/ARE, pmk-1/p38 MAPK, mitochondrial quality control, and protein homeostasis mediated by HSP90AA1. Thirdly, the combined use of multiple methods, such as green extraction with NADES, high-resolution mass spectrometry, transcriptomics, network pharmacology and machine learning, supports phytochemical characterization and target identification. Fourthly, these studies range from *Caenorhabditis elegans* and HepG2/HSF cell models to potential applications in AMD and epidemiological studies of frailty based on hospitals and NHANES. However, the evidence comes largely from invertebrate and cell models or observational studies. Therefore, further work on standardized extracts, dose-response relationships, pharmacokinetics, gut microbiome transformation, and sex-specific effects, together with randomized clinical trials, is required.

We thank all authors for their contributions, the reviewers for rigorous evaluation, and the Frontiers editorial teams for supporting this Research Topic. We hope this Research Topic stimulates further interdisciplinary work at the interface of nutrition, phytochemistry, systems biology, and clinical gerontology.

